# Supplementary material for: The Multivalent Polyampholyte Domain of Nst1, a P-Body-Associated Saccharomyces cerevisiae Protein, Provides a Platform for Interacting with P-Body Components
Source: Int J Mol Sci. 2022 Jul 2;23(13):7380. doi: 10.3390/ijms23137380 (PMC9266425; doi:10.3390/ijms23137380)
Supplement: Supplementary file 1 [file ijms-23-07380-s001.zip › ijms-1761014-supplementary.pdf]

The multivalent polyampholyte domain of Nst1, a P-body-associated *Saccharomyces cerevisiae* protein, provides a platform for interacting with P-body components

Yoon-Jeong Choi [1], Yujin Lee [1], Yuxi Lin [2], Yunseok Heo [2], Young-Ho Lee [2,3,4,5], and Kiwon Song [1,*]

1 Department of Biochemistry, College of Life Science and Biotechnology, Yonsei University, Seoul 03722, Republic of Korea
2 Research Center for Bioconvergence Analysis, Korea Basic Science Institute (KBSI), , Chungbuk 28119, Republic of Korea
3 Department of Bio-Analytical Science, University of Science and Technology (UST), Daejeon 34113, Republic of Korea
4 Graduate School of Analytical Science and Technology (GRAST), Chungnam National University (CNU), Daejeon 34134, Republic of Korea
5 Research Headquarters, Korea Brain Research Institute (KBRI), Daegu 41068, Korea

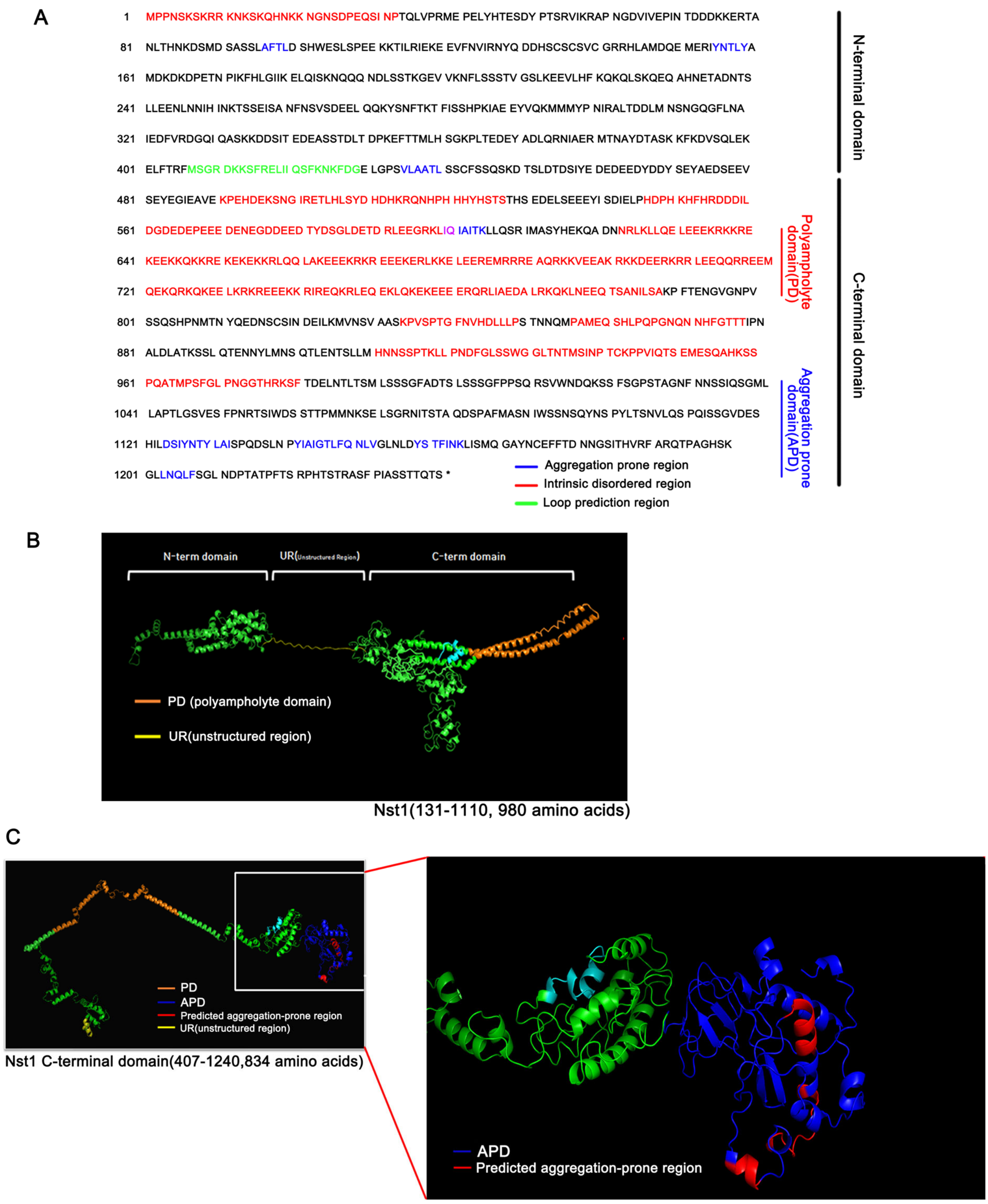

Supplementary Figure S1: The Nst1 amino acid sequence.

(A) The Nst1 sequence is indicated by the predictions described in Figure 1A,B. The loop prediction was derived from a GalaxyWEB [1] prediction of the Nst1 secondary structure. **(B)** A prediction of the Nst1 (131-1110, 980 amino acids) secondary structure by GALAXY WEB[1]. **(C)** A prediction of the Nst1$_{CTD}$ (407-1240, 834 amino acids) secondary structure by GALAXY WEB [1]. **(B-C)** Each predicted domain is represented by a specific color. The polyampholyte domain (PD) is orange, the aggregation-prone domain (APD) is blue, the predicted aggregation-prone region is red, and the unstructured region (UR) is yellow.

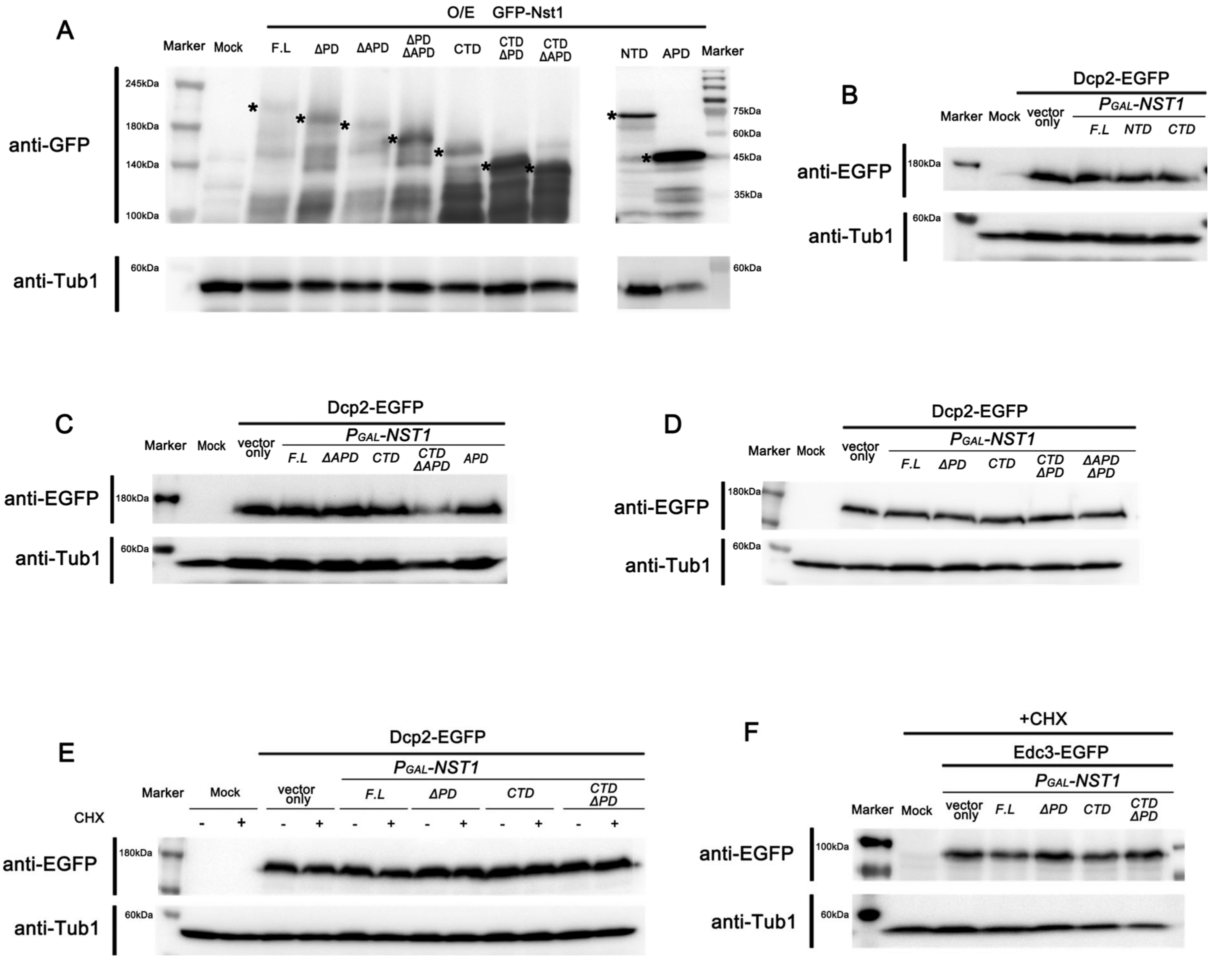

Supplementary Figure S2: Verification of the ectopic expression of various EGFP-tagged Nst1 domain deletion mutants and the endogenous expression of EGFP-Dcp2 and EGFP-Edc3 in cells overexpressing Nst1 domain deletion mutants.

The overexpression of GFP-tagged Nst1 domain deletion mutants was confirmed by Western blotting in each strain collected for fluorescence microscopy, as Figures 2A, 3A, and 4A show. An anti-EGFP antibody (600-101-215 Rockland) was used for Western blots, and the same blot was also probed with anti-Tub1 (T5168, Sigma) as a positive expression control. (A) Ectopic expression of GFP-tagged Nst1 domain deletion mutants in BY4741 wild-type cells (Figures 2A, 3A, and 4A). The detected bands are marked with asterisks. (B–D) Endogenous EGFP-Dcp2 expression in each condition of (B) Figures 2B,C, (C) 3B,C, and (D) 4B,C. (E) Endogenous expression of EGFP-Dcp2 confirmed in the presence and absence of CHX (100 μg/mL) after overexpression of Nst1 domain deletion mutants in Figure 5A,B. (F) Endogenous EGFP-Edc3 expression confirmed in the presence of CHX (100 μg/mL) after overexpression of Nst1 domain deletion mutants in Figures 6A,B.

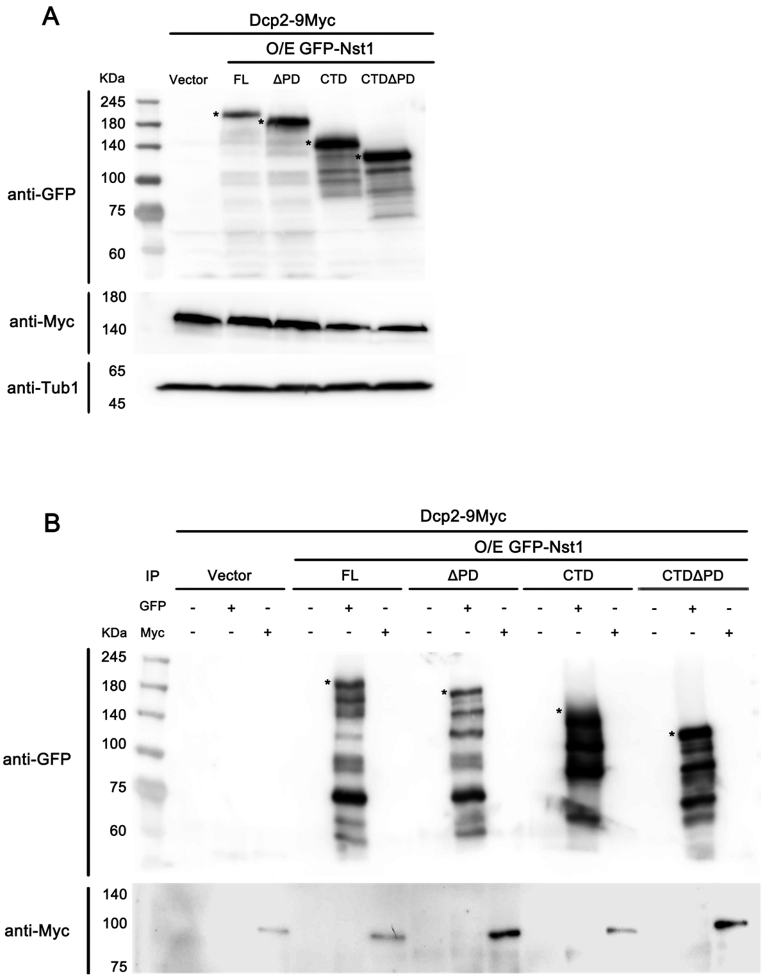

Supplementary Figure S3: Verification of the physical interaction of the overexpressed Nst1 domain deletion mutants and the endogenous Dcp2 by co-immunoprecipitation.

Physical interaction between the overexpressed Nst1, Nst1$_{\Delta PD}$, Nst1$_{CTD}$, Nst1$_{CTD\Delta PD}$ and 9Myc-tagged Dcp2 was examined by co-immunoprecipitation (Co-IP). The GFP-tagged Nst1 domain deletion mutants were transformed to cells endogenously expressing 9Myc-tagged Dcp2 (YSK3592). The presence of the GFP-tagged Nst1 domain deletion mutants and 9Myc-tagged Dcp2 was verified by the Western blot using anti-GFP (600-101-215, Rockland, Limerick, PA, USA) and anti-Myc (#2287, Cell signaling, Danvers, MA, USA). (A) Expression of each *GAL*-induced Nst1 domain deletion mutant and 9Myc-tagged Dcp2 was confirmed by Western blot analysis before cell lysis. The same number of cells overexpressing full-length Nst1, Nst1$_{\Delta PD}$, Nst1$_{CTD}$, Nst1$_{CTD\Delta PD}$ in endogenously expressing 9Myc-tagged Dcp2 were collected. The expression level of Tub1 was confirmed by anti-Tub1 (T5168, Sigma, St. Louis, MO, USA) to clarify a usage of the same amount of protein in each Co-IP analysis. (B) Co-IP analysis of the full-length Nst1, Nst1$_{\Delta PD}$, Nst1$_{CTD}$, Nst1$_{CTD\Delta PD}$ and 9Myc-tagged Dcp2. The protein extract of *GAL*-induced cells overexpressing each Nst1 domain deletion mutant and endogenously expressing Dcp2 was incubated in Dynabeads Protein G (10004D, Thermo Fisher Scientific, Waltham, MA, USA) conjugated with anti-GFP (D153-3, MBL, Woburn, MA, USA) and anti-Myc (#2287, Cell signaling, Danvers, MA, USA) to purify the overexpressed GFP-tagged Nst1 domain deletion mutants and 9Myc-tagged Dcp2, respectively. Dynabeads Protein G without any antibody was used as a negative control.

1. Ko, J.; Park, H.; Heo, L.; Seok, C. GalaxyWEB server for protein structure prediction a nd refinement. *Nucleic Acids Res.* **2012**, *40*, W294-297. https://doi.org/10.1093/nar/gks493.
